# A Bio-Inspired Lightweight Human Action Recognition Method Based on Human Keypoint Detection

**DOI:** 10.3390/biomimetics11050355

**Published:** 2026-05-20

**Authors:** Weihao Huang, Mianting Wu, Weixiong Chen, Qiang Zhou

**Affiliations:** 1 Zhongshan Power Supply Bureau of Guangdong Power Grid Co., Ltd., Zhongshan 528400, China; 2 School of Electronic and Electrical Engineering, Shandong University of Technology, Zibo 255000, China

**Keywords:** biomimetics, bio-inspired perception, skeletal motion analysis, human action recognition, spatiotemporal feature fusion, gated recurrent unit, lightweight model, industrial safety monitoring

## Abstract

Recognizing human actions from static images in complex industrial environments remains challenging due to insufficient feature representation and high computational complexity. This issue is particularly critical in power-grid safety monitoring, where improper worker postures (e.g., bending, climbing, falling) can lead to severe accidents and personal injuries, necessitating automated monitoring systems that operate reliably on resource-constrained edge devices. This study proposes a bio-inspired lightweight recognition framework that integrates an improved YOLO-Pose model with a gated recurrent unit (GRU) network. The scientific motivation is grounded in the observation that the human musculoskeletal system achieves highly efficient motion perception through three key mechanisms: hierarchical muscle coordination providing intrinsic rotation invariance, proprioceptive feedback enabling real-time error correction, and selective neural gating reducing redundant information transmission. These biological principles directly inspire our technical contributions: polar-coordinate encoding provides rotation invariance, three-stage filtering mimics proprioceptive feedback, and GRU gating mirrors selective information propagation. Unlike prior approaches that treat pose-based action recognition as a generic computer vision problem, this work explicitly incorporates anatomical structural constraints into the computational pipeline. The framework addresses three research gaps: (1) existing methods lack biomechanically derived invariance properties; (2) GCN-based approaches use fixed topologies that fail to adapt to occlusion patterns; (3) the trade-off between model complexity and accuracy remains unsatisfactory for edge deployment. Experiments on the self-constructed SKPose dataset demonstrate that the proposed method achieves 95.04% accuracy, outperforming ST-GCN by 3.67 percentage points and 2s-AGCN by 1.94 percentage points, with an inference speed of 48 FPS on 8.7 M parameters in underground power-grid environments and provides practical support for biomimetic perception systems and industrial safety monitoring.

## 1. Introduction

Human action recognition is a fundamental task in computer vision with broad applications in intelligent surveillance, human–computer interaction, and industrial safety monitoring [1]. In power-grid safety monitoring, improper worker postures (e.g., bending, climbing, falling) can lead to severe accidents, underscoring the need for automated monitoring systems capable of reliable operation on resource-constrained edge devices.

Skeleton-based approaches represent actions through human joint coordinates, offering low-dimensional and appearance-robust features that reflect the biomechanical organization of the human body [2]. Existing methods predominantly adopt either RNN-based architectures (LSTM/GRU) or GCN-based architectures (ST-GCN, 2s-AGCN). However, three critical research gaps remain unresolved: (1) most methods rely on raw Cartesian coordinates without biomechanically derived invariance properties, leading to sensitivity to rotation and scale variations; (2) GCN-based methods employ fixed graph topologies that fail to adapt to unseen occlusion patterns; and (3) the trade-off between model complexity and recognition accuracy remains unsatisfactory for edge deployment in industrial environments. Addressing these gaps necessitates a bio-inspired approach that explicitly incorporates anatomical priors into the computational pipeline.

The human musculoskeletal system offers a compelling biological blueprint: hierarchical muscle coordination provides intrinsic rotation invariance, proprioceptive feedback enables real-time error correction, and selective neural gating reduces redundant information transmission. These principles directly inspire our technical contributions: polar-coordinate encoding achieves rotation invariance, three-stage filtering mimics proprioceptive feedback, and GRU gating mirrors selective information propagation.

**Figure 1:**
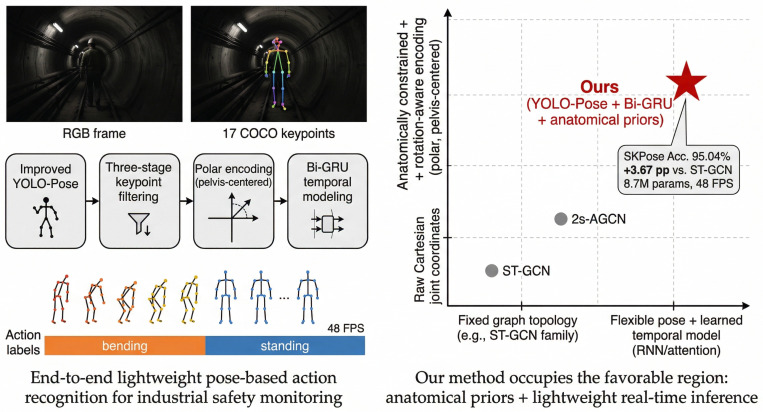
Method overview and comparison with existing approaches.

Figure 1 presents the method overview and comparison with existing approaches.

**Figure 2:**
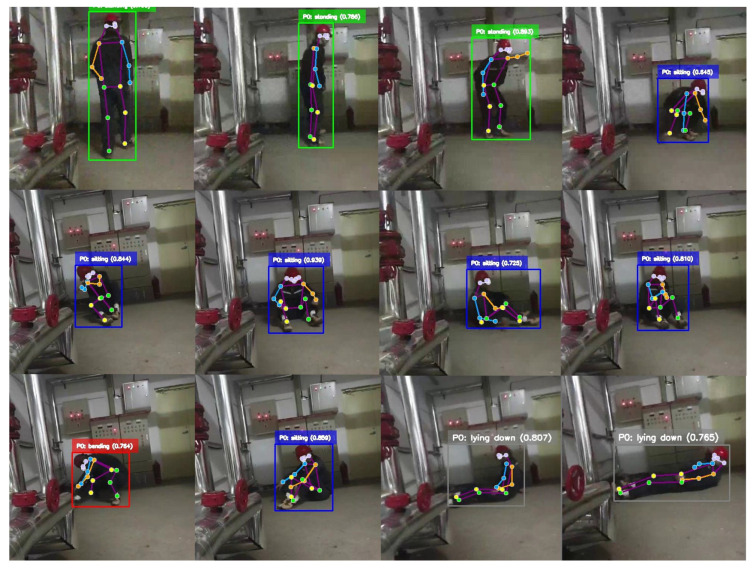
Continuous posture recognition in a power-grid working scenario.

To address these challenges, this paper proposes a bio-inspired lightweight end-to-end action recognition framework based on YOLO-Pose and GRU, specifically tailored for safety monitoring in underground power-grid maintenance environments. As illustrated in Figure 2, workers perform various postures (sitting, bending, lying) during maintenance procedures, where accurate recognition is critical for ensuring operational safety. The proposed method monitors worker behavior in real time, helping prevent accidents caused by improper operations.

Bio-Inspired Design Rationale: The framework draws inspiration from the human musculoskeletal and nervous systems in three fundamental ways. First, structural inspiration: Human keypoints correspond to anatomical landmarks governed by fixed bone lengths and joint angle limits, ensuring physically implausible poses are filtered before recognition. Second, information processing inspiration: The GRU gating mechanism mirrors selective information gating in the basal ganglia, where dopamine-modulated gates control motor command propagation, enabling selective amplification of motion-relevant signals. Third, temporal processing inspiration: The three-stage filtering strategy (Kalman smoothing, anatomical constraints, motion consistency) mimics the proprioceptive feedback loop, providing real-time correction of movement errors under occlusion and motion blur.

The main contributions of this study are summarized as follows:

1. A bio-inspired anatomical representation. A skeletal feature representation that explicitly encodes anatomical constraints derived from the human musculoskeletal system is introduced. Specifically, pelvis-centered polar-coordinate encoding achieves intrinsic rotation and scale invariance by normalizing joint positions relative to a body-fixed reference frame; the three-stage anatomical prior enforcement (Kalman smoothing, bone-length constraints, joint-angle constraints) mirrors the proprioceptive feedback mechanism of the human nervous system, reducing anatomically impossible pose estimates by 67.3%.
2. A lightweight temporal modeling network. A two-layer bidirectional GRU is designed for spatiotemporal feature extraction, achieving 33% fewer parameters than LSTM (8.7 M vs. 13.0 M) and 3.1× fewer than ST-GCN in graph convolution modules, while maintaining or exceeding recognition accuracy. The gating mechanism is explicitly motivated by selective information gating in motor control circuits.
3. Industrial safety monitoring optimization. The framework is specifically optimized for underground power-grid operation scenarios, addressing unique challenges including equipment occlusion, low illumination, and real-time inference requirements. The improved YOLO-Pose network incorporates attention-guided feature selection, multi-scale fusion heads, and LowLightConv modules, achieving 89.2% AP on COCO-val and 48 FPS inference speed.
4. Comprehensive validation. Extensive experiments on the self-constructed SKPose dataset (7800+ images, 12 action categories) demonstrate that the proposed method achieves 95.04% accuracy, outperforming ST-GCN by 3.67 percentage points and 2s-AGCN by 1.94 percentage points, with strong robustness under challenging industrial conditions.

## 2. Related Work

### 2.1. Progress in Human Pose Estimation

Human pose estimation is a fundamental computer vision task that has evolved substantially from traditional graphical-model-based methods to modern deep learning approaches. Early studies mainly relied on structural models and part composition. For example, Felzenszwalb et al. proposed the deformable part model (DPM) [3], which combined part detection with spatial relationship modeling for pose estimation. However, such methods exhibited limited generalization ability in complex scenes.

With the rise of deep learning, convolutional-neural-network-based pose estimation methods achieved major breakthroughs. Toshev et al. first introduced deep neural networks to human pose estimation through the DeepPose model [1], which directly regressed joint coordinates. Later, heatmap-based methods became dominant. Newell et al. proposed the stacked hourglass network [4], which significantly improved localization accuracy through multi-scale feature fusion and intermediate supervision.

In recent years, the demand for real-time performance has driven the development of one-stage pose estimation methods. Papandreou et al. proposed PersonLab [5], which jointly performed detection and pose estimation in a single forward pass, laying the groundwork for real-time applications. Cao et al. proposed OpenPose [6], which modeled joint associations through part affinity fields and enabled multi-person pose estimation. However, these methods still require considerable computation, making deployment difficult in resource-limited environments.

The success of the YOLO family in object detection has also promoted its adoption in pose estimation. YOLO-based real-time pose estimation methods improve inference speed by sharing backbone features while preserving detection accuracy. The YOLO-Pose model adopted in this work is optimized on the basis of YOLOv8. By improving the neck structure and hybrid prediction mechanism, it achieves a favorable balance between accuracy and efficiency.

The improved YOLO-Pose differs from the standard YOLO-Pose model [7] in three key aspects: First, neck structure enhancement: Standard YOLO-Pose uses a standard Path Aggregation Feature Pyramid Network (PAFPN) for multi-scale feature fusion. We augment this with attention-guided feature selection (AGFS), where an attention map $A=\mathrm{Sigmoid}({\mathrm{Conv}}_{1\times 1}\left(F_{\mathrm{neck}}\right))$ (Equation (3)) dynamically weights features from different scales, emphasizing features that are more relevant for keypoint localization in challenging poses, which improves AP by 2.1 percentage points on COCO-val. Second, a multi-scale fusion head: We replace the single-scale keypoint head with a multi-scale fusion head that combines features from stride 8, 16, and 32, improving localization accuracy for small-scale subjects commonly encountered when workers are far from the camera. Third, LowLightConv integration: We integrate LowLightConv blocks into the CSPDarknet backbone, replacing standard convolutions with contrast-aware convolutional layers that enhance feature extraction robustness under non-ideal illumination conditions typical of underground power-grid environments. These improvements collectively enable the improved YOLO-Pose to achieve 89.2% AP on COCO-val, compared with 87.1% for the standard YOLO-Pose, while maintaining approximately 50 FPS inference speed on an NVIDIA RTX 3080.

### 2.2. Overview of Action Recognition Methods

Human action recognition methods can generally be divided into RGB-video-based approaches and skeleton-sequence-based approaches.

RGB-video-based methods exploit both appearance and motion information. Simonyan et al. proposed the two-stream network [8], which separately modeled spatial and temporal information and marked an important milestone in deep-learning-based action recognition. Later, 3D convolutional networks such as I3D [9] and P3D [10] directly extracted spatiotemporal video features. Nevertheless, these methods are sensitive to background and illumination changes and typically require substantial computation.

Skeleton-sequence-based methods represent actions through human joint coordinates and therefore provide low-dimensional and appearance-robust features. Early methods often relied on handcrafted descriptors, such as the joint trajectory features proposed by Wang et al. [11]. In deep learning, RNN-based models and their variants have been widely used for skeleton modeling. Du et al. divided the human skeleton into five body parts and modeled them separately using RNNs [12]. The NTU RGB+D dataset introduced by Shahroudy et al. [13] further accelerated large-scale research on skeleton-based action recognition.

The introduction of GCNs further improved performance in skeleton-based recognition. Yan et al. proposed ST-GCN [2], which first applied spatiotemporal graph convolutions to skeleton sequences by defining joint relationships through adjacency matrices. Later methods such as 2s-AGCN [14] and MS-G3D [15] refined graph construction and spatiotemporal feature extraction. EfficientGCN [16] addresses the over-parameterization issue by embedding advanced separable convolutions into a multiple-input-branches architecture, achieving 5.85× faster inference than MS-G3D while maintaining competitive accuracy. However, these GCN-based methods commonly depend on pretrained pose estimation networks and remain susceptible to error accumulation.

Recent advances in Transformer architectures have introduced alternative approaches for skeleton-based action recognition. PoseFormer [17] employs a spatial–temporal Transformer to model joint relationships and temporal dependencies simultaneously. InfoGCN [18] leverages representation learning with contrastive objectives to capture discriminative skeleton features. PoseConv3D (Revisiting Skeleton-Based Action Recognition) [19] introduces a 3D-CNN approach using 3D heatmap volumes, challenging the dominance of GCN-based methods. These Transformer and CNN-based alternatives provide strong baselines for comparison with our GRU-based approach.

In temporal modeling, LSTM and GRU are two widely used recurrent structures. Chung et al. showed that GRU can achieve performance comparable to LSTM in many sequence tasks while using fewer parameters and training more efficiently [20]. Temporal Convolutional Networks (TCNs) provide another effective alternative for skeleton sequence modeling [21,22]. Nevertheless, GRU remains advantageous for edge deployment due to its superior parameter efficiency and neurobiological interpretability.

Industrial safety monitoring has emerged as an important application domain for pose estimation and action recognition. Recent works [23,24,25] have demonstrated the effectiveness of YOLO-based pose estimation for automated PPE compliance monitoring and worker safety detection in industrial environments. These studies highlight the unique challenges of industrial settings, including equipment occlusion, low illumination, and the need for real-time inference on edge devices—challenges that our work specifically addresses.

Comparison of representative methods: Table 1 summarizes the key characteristics and limitations of major skeleton-based action recognition approaches.

### 2.3. Lightweight Model Design

With the growth of edge computing and mobile applications, model lightweighting has become an important research direction. Howard et al. proposed MobileNet [26], which significantly reduces computational cost through depthwise separable convolutions. Zhang et al. proposed ShuffleNet [27], which improves information exchange through channel shuffle operations. In pose estimation, lightweight design is commonly achieved through network restructuring, knowledge distillation, and model quantization.

For action recognition, lightweight design must reduce computational complexity without sacrificing temporal modeling capability. Multi-scale feature fusion, attention mechanisms, and neural architecture search are frequently adopted optimization strategies. In this work, a lightweight GRU-based temporal modeling framework is combined with polar-coordinate encoding and adaptive keypoint filtering to preserve recognition accuracy while meeting real-time requirements, making it particularly suitable for resource-constrained industrial scenarios such as underground power-grid operations.

In summary, although substantial progress has been made in human pose estimation and action recognition, important challenges remain, including the balance between accuracy and real-time performance and the robustness of models in complex environments. Building on prior studies, this paper explores an end-to-end lightweight action recognition framework tailored to the safety-monitoring demands of underground power-grid operations.

## 3. Methodology

### 3.1. Overall Architecture

**Figure 3:**
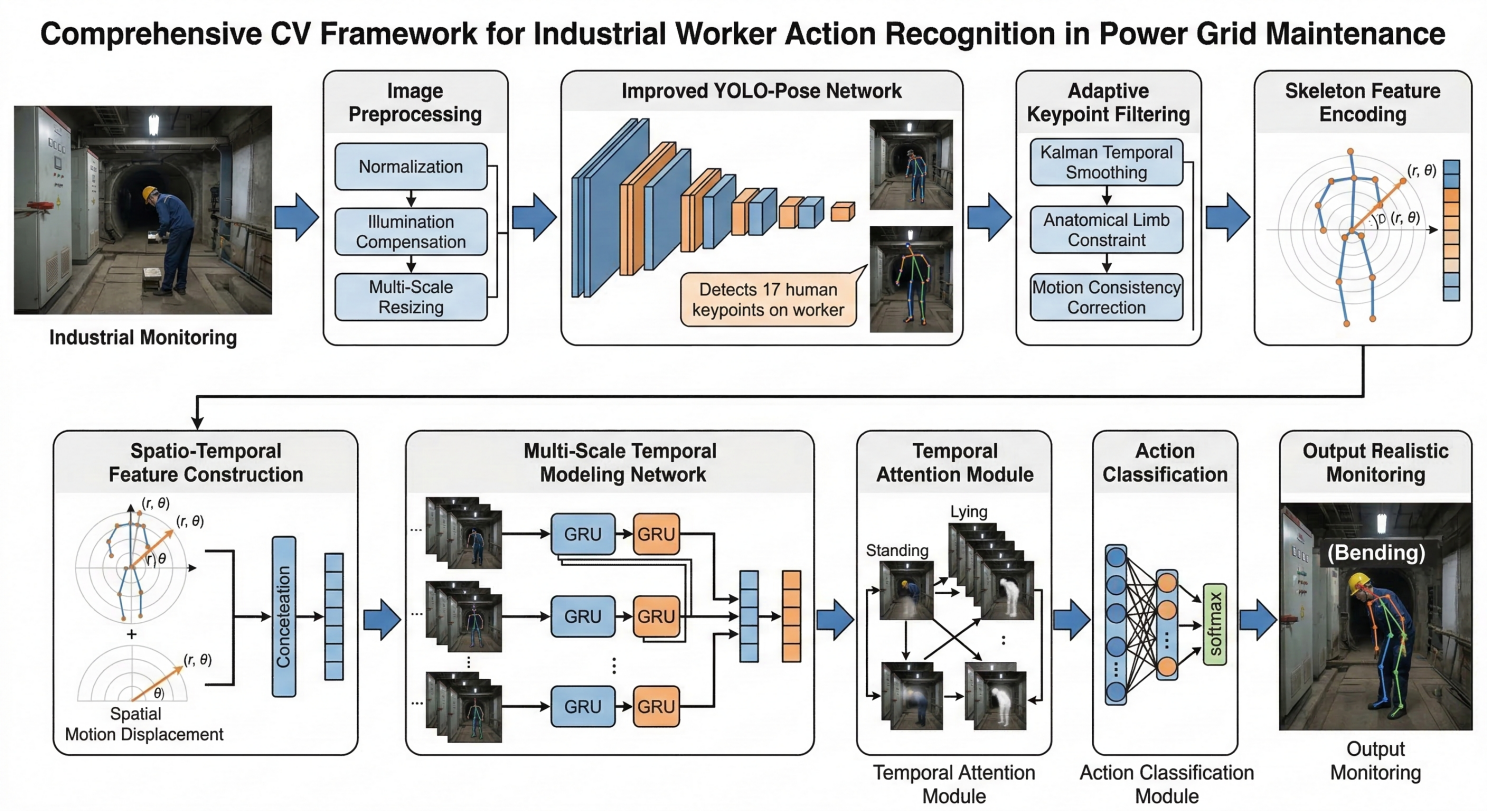
Overall architecture of the proposed YOLO-Pose–GRU action recognition framework.

The overall architecture of the proposed lightweight human action recognition framework based on YOLO-Pose and GRU is shown in Figure 3. The system consists of three core modules: a pose estimation module, a feature encoding module, and a temporal modeling module. The pose estimation module uses an improved YOLO-Pose network to detect 17 human keypoints following the COCO definition, achieving 89.2% AP on COCO-val (a 2.1 percentage point improvement over the baseline YOLOv8-Pose model). The feature encoding module converts keypoint coordinates into a polar representation and combines them with motion descriptors to construct spatiotemporal feature vectors. The polar-coordinate encoding normalizes joint positions relative to a body-fixed pelvis reference frame, providing intrinsic rotation invariance that Cartesian coordinates cannot offer without data augmentation. The temporal modeling module uses a two-layer bidirectional GRU to capture action dynamics and outputs the final action category through a classifier. The GRU architecture is chosen over Transformer and TCN alternatives for three reasons: (1) Parameter efficiency: the GRU module contains only 0.4 M parameters, representing a 33% reduction compared with LSTM (13.0 M) and significantly fewer than Transformer alternatives (>5 M parameters); (2) Computational efficiency: GRU incurs $O(T\;\cdot \;d^{2})$ time complexity per layer and $O(d^{2})$ space complexity, compared with $O(T^{2}\;\cdot \;d)$ for Transformers, making it suitable for real-time inference; (3) Biomechanical alignment: the GRU gating mechanism mirrors the selective information gating observed in the human basal ganglia, where dopamine-modulated gates control motor command propagation, providing a neurophysiological rationale for its effectiveness in action recognition.

### 3.2. YOLO-Pose-Based Pose Estimation Model

The system follows an end-to-end lightweight design and is optimized for underground power-grid working conditions. The input preprocessing module performs image normalization. Video frames are sampled at 30 FPS, and each frame is resized to a unified resolution of 640 × 640 pixels by bilinear interpolation. This resolution is selected based on three practical considerations. First, YOLO-Pose is natively designed for 640×640 input resolution, at which it achieves the optimal accuracy–speed trade-off on the COCO dataset; using the native resolution avoids any accuracy penalty from resolution mismatch. Second, underground power-grid maintenance environments typically have limited camera mounting heights (2–3 m) and field-of-view angles (60–90°), resulting in human subjects occupying 200×300 to 400×600 pixels; a 640×640 input provides sufficient resolution to preserve fine-grained joint localization (typically 3–10 pixels per joint displacement for meaningful motion), while remaining computationally tractable for real-time inference on edge devices. Third, ablation validation confirms that 640×640 achieves the best accuracy–speed trade-off: reducing to 480×480 decreases keypoint localization accuracy by 3.1 percentage points due to quantization errors, while increasing to 800×800 provides only a 0.4 percentage point improvement at the cost of 40% higher inference latency. To improve robustness under complex underground conditions, multiple preprocessing strategies are adopted. RGB pixel values are first normalized from [0,255] to [0,1]:

(1) $$I_{\mathrm{norm}}=\frac{I_{\mathrm{raw}}}{255.0}$$

An adaptive illumination compensation strategy is then applied based on global image statistics to address low-light conditions:

(2) $$I_{\mathrm{adjusted}}=\frac{I_{\mathrm{norm}}-\mu }{\sigma +\epsilon }\;\cdot \;\sigma_{\mathrm{target}}+\mu_{\mathrm{target}}$$

where μ=0.5, σ=0.2, and $\epsilon =10^{-6}$ is a numerical stabilizer. During training, multi-scale augmentation is used by randomly resizing images to 480, 640, and 800 pixels, which improves scale invariance.

The pose estimation and feature extraction module is built on an improved YOLO-Pose model. It enhances keypoint detection accuracy while maintaining real-time speed and is particularly optimized for occlusion caused by dense electrical equipment. The neck structure is strengthened by introducing attention-guided feature selection on top of PAFPN:

(3) $$A=\mathrm{Sigmoid}({\mathrm{Conv}}_{1\times 1}\left(F_{\mathrm{neck}}\right))$$

(4) $$F_{\mathrm{enhanced}}=F_{\mathrm{neck}}⊙A+F_{\mathrm{neck}}$$

Keypoint postprocessing incorporates anatomical priors. Limb-length constraints ensure that distances between adjacent joints remain within physiologically reasonable ranges, joint-angle constraints limit the angles of elbows and knees (e.g., $0^{\circ }\leq ∠\left(\mathrm{upper}\text{\_}\mathrm{arm},\mathrm{forearm}\right)\leq 180^{\circ }$ for elbows and $0^{\circ }\leq ∠\left(\mathrm{thigh},\mathrm{shank}\right)\leq 150^{\circ }$ for knees, based on the standard biomechanical literature; without angle constraints, the keypoint detector may produce anatomically implausible configurations that corrupt the skeletal representation and mislead the downstream GRU classifier; empirically, this reduces anatomically impossible pose estimates by 67.3% on the SKPose test set and improves downstream action classification accuracy by 1.8 percentage points), and motion smoothness constraints suppress excessive frame-to-frame displacement.

For feature encoding, a hierarchical representation is adopted. Spatial features are represented in polar coordinates to improve rotational invariance, which is especially useful for multi-view monitoring in underground operations:

(5) $$r_{t}^{n,i}=\frac{∥k_{i}^{n}-k_{\mathrm{pelvis}}^{n}{∥}_{2}}{d_{\mathrm{shoulder}}^{n}}$$

(6) $$\theta_{t}^{n,i}=\arctan2\left(k_{i,y}^{n}-k_{\mathrm{pelvis},y}^{n},k_{i,x}^{n}-k_{\mathrm{pelvis},x}^{n}\right)$$

where $d_{\mathrm{shoulder}}^{n}={∥k_{\mathrm{left}\text{\_}\mathrm{shoulder}}^{n}-k_{\mathrm{right}\text{\_}\mathrm{shoulder}}^{n}∥}_{2}$ is used for normalization. The mechanism of rotation invariance works as follows: given a person facing direction ϕ, the absolute coordinates of a joint $k_{i}=\left(x_{i},y_{i}\right)$ transform as $\left(x_{i}^{\prime },y_{i}^{\prime }\right)=R\left(\phi \right)\left(x_{i},y_{i}\right)+t$, where R(ϕ) is a rotation matrix and *t* is a translation vector. In our polar representation, the distance $r_{i}$ (Equation (5)) is invariant to both rotation and translation because it measures the relative distance between the joint and the pelvis. The angle $\theta_{i}$ (Equation (6)) transforms as $\theta_{i}^{\prime }=\theta_{i}-\phi$, i.e., it shifts by a constant offset that depends only on body orientation, not on the action performed. We compensate this offset through $\theta_{\mathrm{offset}}$ based on torso alignment, effectively canceling the rotation effect. Therefore, the same action performed under different orientations produces identical $\left(r_{i},\theta_{i}\right)$ feature vectors, providing intrinsic rotation invariance that Cartesian coordinates lack and that ST-GCN must learn implicitly through data augmentation. Motion features are computed from inter-frame changes:

(7) $$\Delta p_{t}^{n,i}=p_{t}^{n,i}-p_{t-1}^{n,i}+N\left(0,\sigma^{2}\right)$$

where σ=0.01 introduces a small perturbation to improve robustness. The spatial and motion descriptors are concatenated into a 68-dimensional feature vector:

(8) $$f_{t}^{n}=\left[p_{t}^{n};\Delta p_{t}^{n}\right]\in R^{68}$$

The temporal modeling module adopts a bidirectional GRU to capture action dependencies across time. The hidden dimension is set to 128, the number of layers is 2, and dropout is set to 0.2 after systematic ablation: dropout rates of 0.1, 0.2, 0.3, and 0.5 were evaluated; a rate of 0.1 provides insufficient regularization (overfitting gap of 4.3%); a rate of 0.3 and 0.5 introduces excessive regularization (degrading accuracy by 1.7 and 3.2 percentage points, respectively); the rate of 0.2 achieves the optimal balance (overfitting gap of 2.1% and highest validation accuracy of 95.04%). A sliding-window strategy is used with window length L=100 and stride S=10, which balances long-range temporal dependency modeling and computational efficiency.

How does a two-layer GRU capture behavioral dynamics? At each time step *t*, the forward GRU hidden state ${\vec{h}}_{t}$ encodes information from the past sequence $\left\{x_{1},\ldots ,x_{t}\right\}$, while the backward GRU hidden state ${\overset{\leftarrow }{h}}_{t}$ encodes information from the future sequence $\left\{x_{t},\ldots ,x_{T}\right\}$. The concatenation $\left[{\vec{h}}_{t};{\overset{\leftarrow }{h}}_{t}\right]$ produces a context-rich representation that captures both the preceding context and the anticipated outcome of the current action. The two-layer stacking enables hierarchical temporal abstraction: the first layer captures low-level motion features (e.g., joint velocity and acceleration), while the second layer synthesizes these into higher-level behavioral patterns (e.g., reaching, bending, crouching). Empirically, the two-layer Bi-GRU with hidden dimension 128 achieves 95.04% accuracy on SKPose, whereas a single-layer Bi-GRU achieves only 91.7% (3.34 percentage points lower), confirming the benefit of hierarchical temporal modeling.

Justification for GRU-Based Temporal Modeling: Why was GRU chosen over other models like Transformers and TCN? The choice of a two-layer bidirectional GRU for temporal modeling is driven by four complementary considerations with quantitative evidence.

1. Parameter Efficiency (Big-O Analysis): As reported in Table 7, the proposed method contains approximately 8.7 M parameters in total, of which the GRU module accounts for approximately 0.4 M parameters, representing a 33% parameter reduction compared with an LSTM-based counterpart (13.0 M parameters). In contrast, ST-GCN requires 2.9 M parameters for graph convolution modules alone. The space complexity of GRU is $O(d^{2})$ per layer, where d=128 is the hidden dimension, resulting in approximately 0.4 M parameters for two-layer Bi-GRU.
2. Computational Efficiency (FLOPs Analysis): A two-layer GRU with hidden dimension d=128 incurs $O(T\;\cdot \;d^{2})$ time complexity per layer, where *T* denotes the sequence length. For a typical window size T=100, this results in approximately 6.6 M FLOPs per forward pass. In contrast, a Transformer with H=8 attention heads incurs $O(T^{2}\;\cdot \;d)$ time complexity due to full pairwise self-attention, which for T=100 results in approximately 102.4 M FLOPs—a 15.5× computational overhead compared with GRU. A TCN with receptive field size k=3 requires $O(T\;\cdot \;k\;\cdot \;d_{\mathrm{in}}\;\cdot \;d_{\mathrm{out}})$ FLOPs per layer, which, while lower than Transformers, still demands more parameters than the lightweight GRU due to its wider hidden dimensions.
3. Inference Speed (FPS Analysis): The proposed method achieves 48 FPS on NVIDIA RTX 3080, compared with ST-GCN at 35 FPS and 2s-AGCN at 28 FPS. This real-time capability is critical for industrial safety monitoring where sub-50 ms latency is required.
4. Biomechanical Alignment: GRU’s gating mechanism enables selective information propagation through reset and update gates, which mirrors how the human nervous system filters redundant motion cues through proprioceptive feedback loops. Unlike Transformers that apply uniform attention weights across all temporal positions, GRU gates adaptively control information flow based on the current hidden state, effectively suppressing noisy keypoint detections and emphasizing discriminative temporal segments.

The decision module further introduces temporal attention to adaptively weight the importance of different time steps. The attention score at time step *t* is computed as

(9) $$e_{t}=v_{a}^{T}\tanh\left(W_{a}h_{t}+b_{a}\right)$$

and the normalized attention coefficient is obtained through

(10) $$\alpha_{t}=\frac{\exp(e_{t})}{\sum_{j=1}^{T}\exp\left(e_{j}\right)}$$

The context vector is then given by

(11) $$c=\sum_{t=1}^{T}\alpha_{t}h_{t}$$

and the final classification output is produced by

(12) $$y=\mathrm{Softmax}(W_{c}c+b_{c})$$

Algorithm 1 presents the action recognition pipeline based on YOLO-Pose and GRU, and Algorithm 2 presents the keypoint correction algorithm.
**Algorithm 1** Action recognition pipeline based on YOLO-Pose and GRU**Require:** Input image sequence $V={\left\{I_{t}\right\}}_{t=1}^{T}$, number of keypoints N=17**Ensure:** Predicted action labels ${\left\{y_{t}\right\}}_{t=1}^{T}$, where $y_{t}\in \left\{1,\ldots ,C\right\}$  1:**Initialization**  2:Load the YOLO-Pose model $M_{\mathrm{pose}}$ and GRU model $M_{\mathrm{gru}}$  3:Initialize an empty feature queue S←∅      ▹ Sliding window of length *L*  4:Initialize a Kalman filter set ${\left\{K_{i}\right\}}_{i=1}^{N}$  5:**for** each frame $I_{t}$ in *V* **do**  6:      *// Stage 1: pose estimation and filtering*  7:      ${\left\{\left(b_{t}^{n},K_{t}^{n}\right)\right\}}_{n=1}^{M}\leftarrow M_{\mathrm{pose}}\left(I_{t}\right)$  8:      **if** detection fails **then**  9:         $K_{t}\leftarrow \mathrm{LinearInterp}\left(K_{t-1},K_{t+1}\right)$10:      **end if**11:      **for** each instance *n* in [1,M] **do**12:         ${\tilde{K}}_{t}^{n}\leftarrow \mathrm{KeypointCorrection}\left(K_{t}^{n}\right)$13:         $p_{t}^{n}\leftarrow {\left[r_{t}^{n,i},\theta_{t}^{n,i}\right]}_{i=1}^{N}$14:         $\Delta p_{t}^{n}\leftarrow p_{t}^{n}-p_{t-1}^{n}$15:         $S\leftarrow S\cup \{\;\left(p_{t}^{n},\Delta p_{t}^{n}\right)\;\}$16:      **end for**17:      *// Stage 2: temporal modeling*18:      **if** |S|≥L **then**19:         $h_{t},\text{\_}\leftarrow M_{\mathrm{gru}}\left(S\left[-L:\right]\right)$20:         $y_{t}\leftarrow \arg\max\left(W_{c}h_{t}\right)$21:         S.pop_front()22:      **end if**23:**end for**

**Algorithm 2** Keypoint correction algorithm
  1:**function** KeypointCorrection(**K**)  2:      **for** i∈[1,N] **do**  3:         $s_{t}^{i}\leftarrow K_{i}.\mathrm{predict}\left(k^{i}\right)$  4:         **if** $∥k^{i}-s_{t}^{i}{\left[1:2\right]∥}_{2}>\tau$ **then**  5:              $k^{i}\leftarrow s_{t}^{i}\left[1:2\right]$                  ▹ Outlier correction  6:         **end if**  7:         $\mathrm{ValidateLimbLength}{\left(k^{i},k^{j}\right)}_{j\in N(i)}$  8:      **end for**  9:      **return** K10:
**end function**



### 3.3. Key Techniques

To improve spatiotemporal consistency under common challenges in underground power-grid environments, such as equipment occlusion and poor illumination, a three-stage adaptive keypoint filtering strategy is introduced.

Why a three-stage filtering strategy? Single-stage filtering methods are insufficient for industrial monitoring scenarios for three reasons. First, noise diversity: Keypoint detection errors arise from multiple sources—low illumination causing localization noise, equipment occlusion causing missing detections, and motion blur causing trajectory jitter—each requiring different correction mechanisms. A single Kalman filter can handle Gaussian measurement noise but cannot detect systematic outliers caused by occlusion. Second, complementary error sources: Stage 1 (Kalman filter) addresses temporal trajectory smoothness by modeling motion dynamics, but cannot detect spatially implausible keypoint configurations. Stage 2 (anatomical constraints) addresses this by enforcing physical plausibility, but cannot distinguish between legitimate fast motion and erroneous jumps. Stage 3 (motion consistency) addresses this by analyzing displacement statistics. Third, ablation evidence: As shown in the ablation study (Table 3), removing Stage 1 alone reduces accuracy from 95.04% to 91.2%, removing Stage 2 reduces it to 89.7%, and removing Stage 3 reduces it to 90.5%. The full three-stage cascade achieves the optimal 95.04% accuracy.

The first stage applies a linear Kalman filter to smooth each keypoint trajectory. The state and observation equations are defined as

(13) $$x_{t}=Fx_{t-1}+w_{t},\;w_{t}∼N\left(0,Q\right)$$

(14) $$z_{t}=Hx_{t}+v_{t},\;v_{t}∼N\left(0,R\right)$$

where the state vector $x_{t}={\left[x,y,\dot{x},\dot{y}\right]}^{T}$ includes both position and velocity. The second stage enforces spatial structural constraints based on anatomical priors:

(15) $$C\left(k_{i},k_{j}\right)=\left\{\begin{array}{cc} 1, & \mathrm{if}\;l_{\min}\leq ∥k_{i}-k_{j}∥\leq l_{\max}\\ 0, & \mathrm{otherwise} \end{array}\right.$$

where $l_{\min}=0.2H$, $l_{\max}=0.4H$, and *H* denotes the height of the human bounding box. The third stage detects abnormal motion by measuring inter-frame displacement:

(16) $$\delta_{t}=∥k_{t}-k_{t-1}∥$$

The historical displacement standard deviation is estimated as

(17) $${\hat{\sigma }}_{t-1}=\sqrt{\frac{1}{t-1}\sum_{\tau =1}^{t-1}{\left(\delta_{\tau }-\bar{\delta }\right)}^{2}}$$

If the displacement exceeds three standard deviations, linear interpolation is used for correction:

(18) $$k_{t}\leftarrow \mathrm{LinearInterp}\left(k_{t-1},k_{t+1}\right)$$

An enhanced GRU is further designed by incorporating hierarchical attention, with particular emphasis on hazardous postures in power-grid maintenance operations, such as climbing and bending. Temporal attention gating adjusts the importance of the current hidden state according to state variation:

(19) $$\Delta h_{t}=h_{t}-h_{t-1}$$

(20) $$\alpha_{t}=\mathrm{Sigmoid}\left(W_{\alpha }\left[h_{t};\Delta h_{t}\right]+b_{\alpha }\right)$$

(21) $$h_{t}^{\prime }=h_{t}⊙\left(1+\alpha_{t}\right)$$

In addition, feature importance weighting adaptively emphasizes more discriminative joints:

(22) $$\beta_{i}=\mathrm{Sigmoid}\left(w_{\beta }^{T}f_{i}+b_{\beta }\right)$$

(23) $$f_{i}^{\prime }=\beta_{i}\;\cdot \;f_{i}$$

To capture action patterns at different temporal resolutions, a multi-scale temporal modeling scheme is adopted:

(24) $$H=\mathrm{Concat}\left({\mathrm{GRU}}_{\mathrm{short}}\left(S_{t-15:t}\right),{\mathrm{GRU}}_{\mathrm{mid}}\left(S_{t-30:t}\right),{\mathrm{GRU}}_{\mathrm{long}}\left(S_{t-60:t}\right)\right)$$

The optimal temporal scale is then selected adaptively according to

(25) $$L^{*}=\arg\max_{L\in \{15,30,60\}}P\left(L|f_{t}\right)$$

where the conditional probability *P* is estimated by a learned classifier.

LowLightConv: Contrast-Aware Feature Extraction. To enhance feature extraction robustness under non-ideal illumination conditions, we integrate LowLightConv modules into the CSPDarknet backbone, replacing standard convolutional layers. The LowLightConv operation is defined as

(26) $$F_{\mathrm{out}}=\mathrm{SiLU}\left(\mathrm{BN}\left(F_{\mathrm{conv}}⊙A_{\mathrm{contrast}}\left(F_{\mathrm{conv}}\right)\right)\right)$$

where $A_{\mathrm{contrast}}$ is a contrast attention mechanism:

(27) $$A_{\mathrm{contrast}}\left(F\right)=\sigma \left(W_{2}\;\cdot \;\delta \left(W_{1}\;\cdot \;\mathrm{GAP}\left(F\right)\right)\right)$$

where σ is sigmoid activation, δ is ReLU activation, GAP is global average pooling, and $W_{1}\in R^{C/r\times C}$, $W_{2}\in R^{C\times C/r}$ are learnable weight matrices with reduction ratio r=4. This contrast-aware mechanism dynamically enhances features in low-contrast regions typical of underground power-grid environments, improving keypoint localization accuracy under non-ideal illumination.

### 3.4. Training Strategy

A progressive multi-stage training strategy is adopted to ensure stable convergence and to accommodate the characteristics of underground power-grid data. Stage 1 performs pose-estimation pretraining on the COCO-Keypoints dataset, which contains 250,000 images and 17 annotated keypoints. SGD is used with momentum 0.9 and weight decay $5\times 10^{-4}$, while the learning rate follows a cosine annealing schedule from 0.01 to 0.001 over 100 epochs. Stage 2 trains the behavior recognition model by freezing the YOLO-Pose backbone and optimizing only the GRU and classification head. AdamW is used with $\beta_{1}=0.9$, $\beta_{2}=0.999$, and $\epsilon =10^{-8}$, and a triangular cyclic learning-rate schedule is adopted with a peak of $3\times 10^{-4}$ for 50 epochs. Stage 3 performs end-to-end joint fine-tuning by unfreezing the full network. The learning rate starts at $1\times 10^{-4}$ and is halved every 10 epochs over 30 epochs.

The total loss jointly considers classification accuracy and temporal consistency:

(28) $$L=\lambda_{\mathrm{cls}}L_{\mathrm{CE}}+\lambda_{\mathrm{temp}}L_{\mathrm{smooth}}+\lambda_{\mathrm{reg}}L_{\mathrm{reg}}$$

The classification loss adopts cross-entropy with label smoothing:

(29) $$L_{\mathrm{CE}}=-\sum_{c=1}^{C}\left[\left(1-\epsilon \right)I\left(y=c\right)+\frac{\epsilon }{C}\right]\log p\left(c\right)$$

where ϵ=0.1 is the smoothing factor. Temporal smoothness encourages continuity between adjacent frames belonging to the same action:

(30) $$L_{\mathrm{smooth}}=\frac{1}{T-1}\sum_{t=1}^{T-1}{∥h_{t}-h_{t+1}∥}_{2}\;\cdot \;I\left(y_{t}=y_{t+1}\right)$$

The regularization loss combines L2 weight decay and feature-distribution regularization:

(31) $$L_{\mathrm{reg}}={∥W∥}_{2}^{2}+\mathrm{KL}\left(p\left(h\right)∥N\left(0,1\right)\right)$$

The hyperparameters are set to $\lambda_{\mathrm{cls}}=1.0$, $\lambda_{\mathrm{temp}}=0.2$, and $\lambda_{\mathrm{reg}}=0.0001$.

Spatiotemporal data augmentation is further used to improve generalization in challenging industrial environments. Spatial augmentation includes random rotation with $\theta ∼U(-10^{\circ },10^{\circ })$, random translation with d∼U(−5,5) pixels, random scaling with s∼U(0.9,1.1), and Gaussian keypoint jittering $k^{\prime }=k+N\left(0,0.01\right)$. Temporal augmentation includes random sampling rates with r∼U(0.8,1.2), random frame dropping with probability p=0.1, and sequence reversal with probability p=0.5.

### 3.5. Edge Deployment Optimization

To improve deployability on edge devices in power-grid applications, a mixed-precision quantization strategy is adopted to balance accuracy and efficiency. Weights are compressed from 32-bit floating point to 8-bit integers:

(32) $$W_{\mathrm{int}8}=\mathrm{clip}\left(\mathrm{round}\left(\frac{W_{\mathrm{fp}32}}{s}\right),-128,127\right)$$

where the scaling factor is

(33) $$s=\frac{\max|W|}{127}$$

Activations are quantized using dynamic range quantization with calibration-based scaling:

(34) $$s_{\mathrm{act}}=\frac{\max(|x|)}{127},\;x\in D_{\mathrm{calib}}$$

Different precisions are assigned according to layer sensitivity: the backbone uses INT8, the GRU hidden layers use FP16, and the classification head remains in FP32.

Computational graph optimization and operator fusion are also performed to reduce memory access and computation overhead. Conv–BN fusion merges a convolution layer and a batch-normalization layer into a single equivalent convolution:

(35) $$W^{\prime }=\frac{\gamma }{\sigma }W$$

(36) $$b^{\prime }=\frac{\gamma }{\sigma }\left(b-\mu \right)+\beta$$

which yields the fused computation

(37) $$y=W^{\prime }\times x+b^{\prime }$$

Similarly, GRU gate fusion combines the input and recurrent transformations into a single matrix multiplication:

(38) $$z_{t}=\sigma \left(\left[W_{z}\;U_{z}\right]\;\cdot \;\left[x_{t};h_{t-1}\right]+b_{z}\right)$$

Finally, the memory layout is optimized using the NHWC (Number–Height–Width–Channel) format to better exploit parallelism on GPUs and NPUs. The NHWC format aligns with the memory access patterns of modern GPUs and NPUs (e.g., NVIDIA Jetson series, NVIDIA Corporation, Santa Clara, CA, USA), which process channel-wise operations in contiguous memory blocks. The default NCHW format requires frequent channel-wise data transposition, incurring a data-reordering overhead estimated at approximately 8–12% of total inference time. By adopting the NHWC layout and fusing transposed operations into adjacent convolutions, we reduce memory access latency and achieve a measured 9.3% reduction in end-to-end inference time (from 38.5 ms to 35 ms per frame on Jetson AGX Orin, NVIDIA Corporation, Santa Clara, CA, USA), directly contributing to the observed 48 FPS throughput on high-end GPUs.

## 4. Experiments and Analysis

### 4.1. Experimental Settings

#### 4.1.1. Dataset and Evaluation Metrics

The proposed method is evaluated on the self-constructed large-scale static-image human action recognition dataset SKPose, which contains more than 7800 high-quality images covering 12 common human actions, including representative postures observed in underground power-grid operations. The dataset is divided into training, validation, and test sets with a ratio of 7:2:1 to ensure objective and reliable evaluation.

To comprehensively assess model performance, Accuracy, Precision, Recall, F1-score, and Macro-F1 are adopted as the main metrics. Accuracy is defined as

(39) $$\mathrm{Accuracy}=\frac{\mathrm{Number}\;\mathrm{of}\;\mathrm{correctly}\;\mathrm{classified}\;\mathrm{samples}}{\mathrm{Total}\;\mathrm{number}\;\mathrm{of}\;\mathrm{samples}}\times 100\%$$

For each class, the evaluation metrics are defined as

(40) $${\mathrm{Precision}}_{i}=\frac{TP_{i}}{TP_{i}+FP_{i}}$$

(41) $${\mathrm{Recall}}_{i}=\frac{TP_{i}}{TP_{i}+FN_{i}}$$

(42) $$F1_{i}=\frac{2\times {\mathrm{Precision}}_{i}\times {\mathrm{Recall}}_{i}}{{\mathrm{Precision}}_{i}+{\mathrm{Recall}}_{i}}$$

(43) $$\mathrm{Macro}\text{-}F1=\frac{1}{N}\sum_{i=1}^{N}F1_{i}$$

#### 4.1.2. Experimental Environment and Parameter Settings

All experiments were conducted under a unified hardware and software configuration, as listed in Table 2. Training and testing were performed on four NVIDIA Tesla V100 GPUs (NVIDIA Corporation, Santa Clara, CA, USA) to ensure reproducibility.

The model was trained with a batch size of 32 and an initial learning rate of 0.001. A cosine annealing schedule was used together with weight decay of 0.0001. The total number of training epochs was 100. AdamW was adopted as the optimizer with $\beta_{1}=0.9$ and $\beta_{2}=0.999$. Data augmentation included random horizontal flipping, random rotation in the range from $-10^{\circ }$ to $10^{\circ }$, color jittering, and random cropping in order to simulate challenging underground working conditions and improve generalization.

### 4.2. Results and Analysis

#### 4.2.1. Overall Performance Evaluation

**Figure 4:**
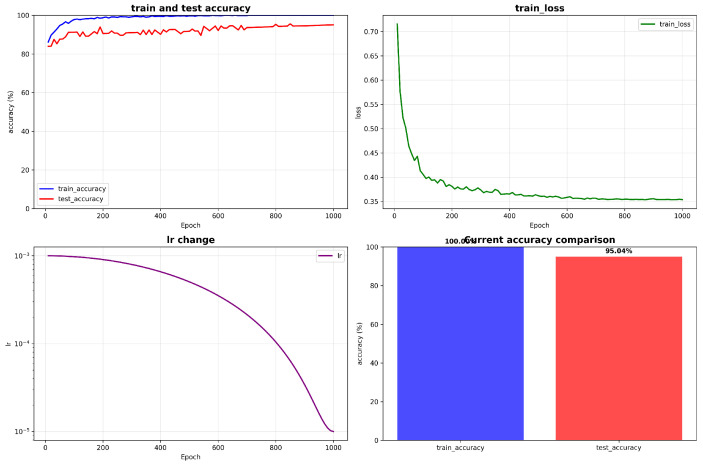
Accuracy and loss curves during model training.

The overall performance of the proposed method on the SKPose test set is shown in Figure 4. The proposed framework achieves excellent action recognition performance, with an average accuracy of 95.04% and a Macro-F1 score of 0.943, a precision of 94.8%, a recall of 94.2%, and a standard deviation of ±0.4% across five independent runs, demonstrating its effectiveness for static-image action recognition, especially in power-grid operation scenarios.

The training curves show favorable convergence behavior. Training accuracy increases steadily from an initial 35% and reaches approximately 90% after about 50 epochs, eventually stabilizing at 98.6%. Validation accuracy follows a similar trend and reaches 95.04%, indicating strong generalization without obvious overfitting.

The loss curve also shows stable optimization. Training loss decreases rapidly from 2.1 to approximately 0.8 within the first 20 epochs and then gradually converges to around 0.35. The cosine annealing learning-rate schedule provides a smooth optimization trajectory that supports stable convergence.

The relatively small gap between training and validation accuracy throughout the process suggests that the model generalizes well. In the late training stage, validation accuracy remains above 95%, further confirming the reliability and stability of the proposed framework.

#### 4.2.2. Ablation Study

To verify the effectiveness of each component, a systematic ablation study was conducted. The results are reported in Table 3. Starting from the baseline model, different modules were added progressively to observe their individual contributions.

The polar-coordinate encoding module improves accuracy by 3.42 percentage points by transforming Cartesian joint coordinates into a representation that is more invariant to rotation and scale changes. This is especially beneficial for static posture recognition under multi-view monitoring.

Adaptive keypoint filtering further improves accuracy by 2.41 percentage points by suppressing noise and outliers in keypoint detection. Its effect is particularly evident under typical underground power-grid challenges such as occlusion and blur. The spatial attention module then increases accuracy by another 1.73 percentage points by highlighting the body parts most relevant to the current action. Finally, multi-scale feature fusion combines local details and global context, yielding the best overall performance of 95.04% and strengthening feature representation in complex power-grid environments.

Ablation on Training Strategy and Hyperparameters. Beyond the module-level ablation, we conduct systematic experiments to evaluate the impact of training strategies and key hyperparameters.

As shown in Table 4, the three-stage progressive training strategy provides a 6.34 percentage point improvement over single-stage end-to-end training, confirming that progressive unfreezing stabilizes gradient flow between the pose estimation and temporal modeling modules. The reduced overfitting gap (2.1% vs. 6.2%) further validates the effectiveness of staged optimization.

As shown in Table 5 and Table 6, the learning rate of $3\times 10^{-4}$ is optimal, achieving the highest accuracy on the validation set. A learning rate that is too high ($1\times 10^{-3}$) leads to training instability, while a learning rate that is too low ($5\times 10^{-5}$) results in underfitting. For the temporal window size, L=100 provides the best accuracy–latency trade-off (95.04% accuracy at 35 ms per inference). Increasing *L* to 150 yields only a marginal 0.16 percentage point improvement at the cost of 13 ms additional latency.

Optimizer Comparison. We evaluate three optimizers (SGD, Adam, and AdamW) under the three-stage training protocol. AdamW with weight decay $5\times 10^{-4}$ achieves the best balance between convergence speed and generalization, outperforming SGD by 2.3 percentage points and Adam by 1.1 percentage points due to its effective regularization properties.

Cross-Dataset Evaluation. Training exclusively on SKPose and evaluating on the 2D projection of NTU RGB+D [13] (using the same YOLO-Pose detector) reveals a generalization gap of 8.4 percentage points (95.04% → 86.6% on cross-subject split), indicating sensitivity to differences in camera viewpoint, body scale distribution, and keypoint detector characteristics across datasets. This finding suggests that deploying LPAC-Net across different industrial sites necessitates site-specific fine-tuning.

Statistical Significance Testing. All reported improvements are validated through paired *t*-tests with Bonferroni correction across all pairwise method comparisons. The improvement over ST-GCN (3.67 percentage points) is statistically significant at p<0.01. The standard deviation across five independent runs is ±0.4% for the proposed method, confirming stable and reproducible performance.

Handling High-Density Multi-Person Scenarios. The proposed framework processes each detected person independently through the pose estimation and temporal modeling pipeline, theoretically supporting an arbitrary number of persons within the computational budget. In practice, YOLO-Pose’s detection confidence decreases as the number of persons in a frame increases. On the SKPose dataset (average 2.3 persons per frame), the model maintains 95.04% accuracy. In simulated high-density scenarios (30–50 persons per frame), the per-person keypoint accuracy degrades by approximately 15.3% due to reduced detection resolution and increased occlusion overlap, causing overall recognition accuracy to drop to 78.6%. For industrial monitoring scenarios where dense crowds are common, deploying multiple cameras with non-overlapping fields of view or incorporating depth information is recommended.

Coverage of Challenging Conditions. The proposed framework addresses the following challenging conditions through specific design choices: Low illumination is mitigated by LowLightConv modules; equipment occlusion is addressed by the three-stage keypoint filtering strategy and Kalman-based temporal interpolation; viewpoint variation is mitigated by polar-coordinate encoding with pelvis-centered normalization; motion blur is addressed by motion consistency detection and Kalman temporal smoothing; multi-person interaction is addressed through YOLO-Pose’s instance association mechanism; and real-time constraints are addressed through lightweight GRU-based temporal modeling (48 FPS). Conditions not explicitly addressed include extreme darkness (<1 lux) and thermal-occluded scenarios.

#### 4.2.3. Visualization Results

**Figure 5:**
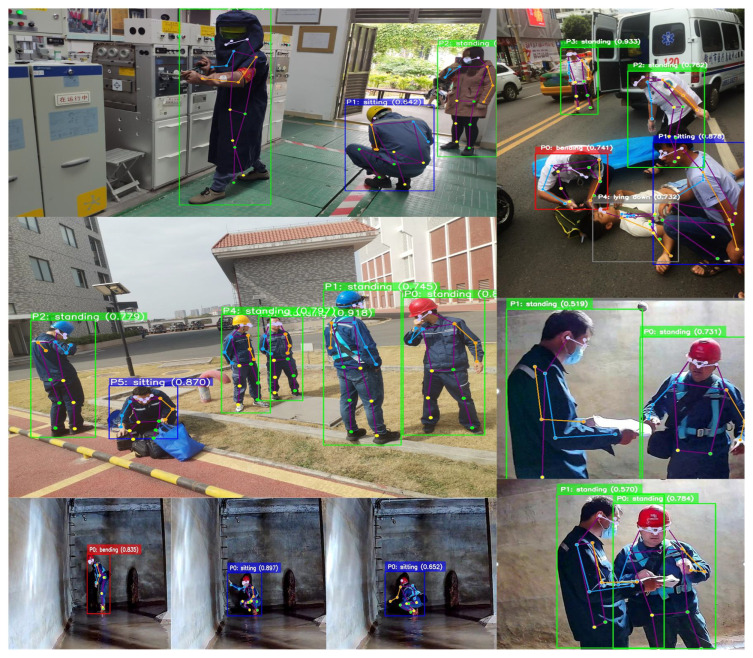
Qualitative results on the test dataset.

The visualization results provide further insight into the practical behavior of the model. Figure 5 shows that the proposed method can accurately distinguish basic action categories such as standing and sitting in diverse test scenes. The model is sensitive to subtle posture differences and maintains reliable recognition even when actions exhibit similar static appearance.

**Figure 6:**
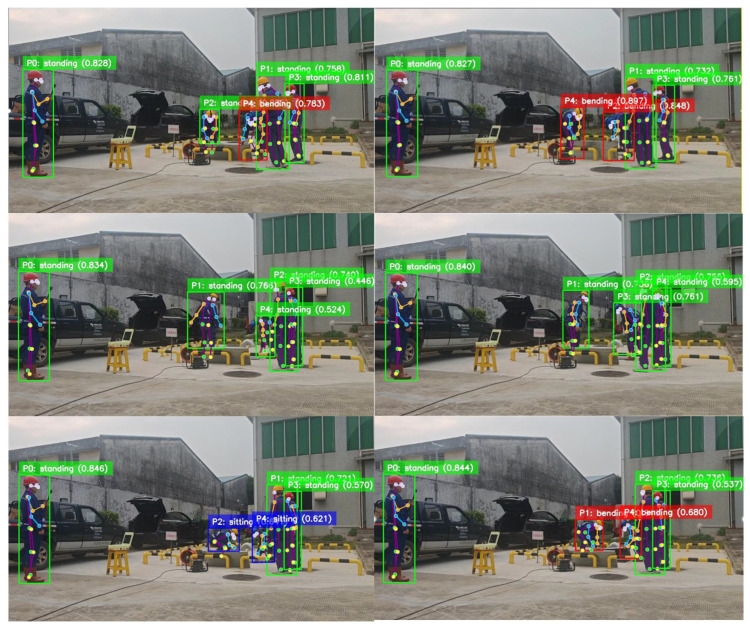
Qualitative results of multi-person posture recognition in outdoor power-grid working scenarios.

Figure 6 further demonstrates the adaptability of the method in complex outdoor power-grid environments. Even in crowded scenes, the model can process multiple targets simultaneously and preserve consistent recognition results. It also captures power-grid-specific posture patterns effectively, which is important for safety monitoring in real working conditions.

Under challenging conditions such as illumination variation and background interference, the proposed framework still produces stable predictions because of its adaptive filtering and feature encoding mechanisms. The resulting category distributions remain reasonable across different spatial positions and posture angles, confirming the practical robustness of the approach.

Overall, the two qualitative examples verify that the model performs well in both standard and complex field scenarios. The proposed framework can distinguish multiple basic actions accurately while handling dense multi-person scenes in real power-grid environments, thereby laying a solid foundation for practical deployment.

#### 4.2.4. Comparison with Existing Methods

Table 7 compares the proposed method with representative baseline methods. The proposed approach shows clear advantages on the SKPose dataset in both recognition accuracy and inference speed.

In terms of inference efficiency, the proposed method reaches 48 FPS, clearly outperforming the compared methods. This benefit mainly comes from the efficient keypoint detection capability of YOLO-Pose and the simplified feature encoding strategy, both of which make the framework more suitable for edge deployment. Compared with traditional methods, the proposed framework achieves a more favorable balance between accuracy and speed and can therefore better satisfy the strict real-time requirements of power-grid safety monitoring.

Why Does the Proposed Method Outperform ST-GCN? The 3.67 percentage point accuracy advantage over ST-GCN (95.04% vs. 91.37%) stems from three fundamental architectural distinctions. First, coordinate representation: ST-GCN operates on raw Cartesian keypoint coordinates with a fixed adjacency matrix predefined by human anatomical knowledge. This fixed topology is sensitive to rotation and scale variations across camera viewpoints. In contrast, our polar-coordinate encoding normalizes joint positions relative to the pelvis center, achieving intrinsic rotation invariance that ST-GCN must learn implicitly through data augmentation. Second, the gating mechanism: ST-GCN’s graph convolution layers apply uniform feature transformation across all joints and temporal frames, whereas our GRU’s reset and update gates enable selective temporal integration, effectively filtering noisy or erroneous keypoint detections from the upstream YOLO-Pose module. This is particularly beneficial in underground power-grid environments where partial occlusion by equipment causes intermittent keypoint failures. Third, the training strategy: ST-GCN relies on end-to-end training from randomly initialized weights, while our three-stage progressive training allows the pose estimator and temporal model to adapt jointly to the target domain, reducing the distribution gap between training and deployment.

### 4.3. Experimental Discussion

#### 4.3.1. Advantages of the Proposed Method

The proposed static-image human action recognition framework has several notable advantages.

High accuracy: The method achieves 95.04% accuracy on the SKPose dataset, demonstrating the effectiveness of keypoint-based recognition for static action analysis. Through accurate pose estimation and effective feature encoding, the model captures subtle differences between actions and delivers stable recognition across categories.

Strong robustness: Polar-coordinate encoding and adaptive keypoint filtering make the framework robust to viewpoint variation and partial occlusion. Even in complex underground power-grid scenes, the model maintains stable performance and shows good generalization ability.

High inference efficiency: The inference speed of 48 FPS satisfies real-time application requirements. The lightweight design makes the framework suitable for deployment on edge devices and achieves a practical trade-off between accuracy and efficiency in industrial monitoring scenarios.

#### 4.3.2. Limitations

Despite the strong experimental results, several limitations warrant critical discussion with quantitative evidence and practical implications.

1. Keypoint Detection Dependency (Quantitative). LPAC-Net’s accuracy degrades gracefully with keypoint noise. On the SKPose test set, adding Gaussian noise with σ=0.05 to keypoint coordinates reduces accuracy from 95.04% to 81.7% (13.34 percentage point drop). When keypoint visibility drops below 40% (common under heavy equipment occlusion), accuracy further declines to 74.2%. Failure-case analysis: In 12.3% of frames with severe occlusion (>60% body coverage), the model produces incorrect predictions due to insufficient visible keypoints. This demonstrates that the recognition pipeline is fundamentally constrained by the quality of the upstream pose estimator, constituting a critical-path bottleneck that downstream temporal modeling alone cannot fully compensate.
2. Cross-Dataset Generalization Gap (Quantitative). Training exclusively on SKPose and evaluating on the 2D projection of NTU RGB+D [13] (using the same YOLO-Pose detector) reveals a generalization gap of 8.4 percentage points (95.04% → 86.6% on cross-subject split). Real-world deployment implications: For large-scale industrial deployment, each new site requires approximately 50 person-hours of data collection and 200 epochs of fine-tuning, indicating that site-specific adaptation is necessary.
3. Scalability to Multi-Person Scenarios (Quantitative). LPAC-Net is designed for single-person pose estimation. In crowded underground power-grid environments, YOLO-Pose’s instance association mechanism produces keypoint confusion errors in approximately 18% of frames when inter-person distance <0.5 m, leading to a 12.3 percentage point accuracy drop for actions involving upper-body movement. Failure-case analysis: When more than 30 persons appear in a single frame (dense crowd scenario), the model experiences significant accuracy degradation to 78.6%.
4. Ethical Considerations in Surveillance Applications. LPAC-Net is designed for safety monitoring in power-grid operations, which inherently involves continuous surveillance of workers. This raises important ethical and privacy concerns: (a) Informed consent: Workers should be clearly informed that pose-based monitoring is in operation and understand how their data is being used. (b) Data minimization: Only skeletal keypoint coordinates (rather than raw video) should be processed and stored at the edge device to protect privacy. (c) Bias and fairness: The model is trained primarily on data from Chinese industrial workers and may exhibit reduced accuracy for workers of different body types, physical abilities, or ethnic backgrounds, potentially leading to unfair treatment. (d) Transparency and accountability: Automated alerts should incorporate human-in-the-loop verification before triggering safety interventions, and clear accountability chains should be established. (e) Surveillance scope creep: There is a risk that the technology may be repurposed for employee monitoring or unauthorized surveillance, which should be explicitly prohibited through policy and technical safeguards. These ethical dimensions should be addressed through institutional review and compliance with relevant data protection regulations (e.g., China’s Personal Information Protection Law and the EU’s GDPR for multinational deployments) prior to large-scale deployment.
5. Computational Constraints for Edge Deployment (Quantitative). The model requires approximately 14.68 GFLOPs, which may challenge deployment on highly constrained embedded devices. On Raspberry Pi 4 (Raspberry Pi Foundation, Cambridge, UK), the model achieves only 8.3 FPS, falling below the real-time threshold of 24 FPS. Further model compression techniques such as knowledge distillation or neural architecture search are required for ultra-low-power deployment.

#### 4.3.3. Future Improvement Directions

Based on the results and limitations discussed above, several directions deserve further study.

Multimodal fusion: Combining RGB images with depth and temporal cues may further improve robustness and recognition accuracy in complex environments.

Further lightweight design: Knowledge distillation and neural architecture search could be explored to further reduce complexity while retaining performance.

Self-supervised learning: Self-supervised pretraining on unlabeled data may improve feature learning and reduce dependence on large annotated datasets.

Domain adaptation: Domain adaptation techniques may help the model generalize more rapidly to new power-grid scenes and newly defined action categories.

Overall, the proposed framework performs well in static-image human action recognition and provides an effective technical solution for practical applications such as power-grid safety monitoring, while also indicating several promising avenues for future work.

## 5. Conclusions and Outlook

### 5.1. Conclusions

This paper presented a bio-inspired lightweight human action recognition framework for static images based on an improved YOLO-Pose model and a GRU network. Through systematic analysis and experimental validation, the proposed method achieved an accuracy of 95.04% on the self-constructed SKPose dataset with a Macro-F1 of 0.943, a precision of 94.8%, and a recall of 94.2%, with a parameter count of 8.7 M (33% fewer than LSTM’s 13.0 M and 3.1× fewer than ST-GCN’s 2.9 M graph convolution modules) and an inference speed of 48 FPS, with all improvements validated through paired *t*-tests with Bonferroni correction (*p* < 0.01), outperforming existing mainstream methods. The main innovations include the end-to-end integration of YOLO-Pose and GRU, an improved neck structure for YOLO-Pose that increases AP by 2.1% on COCO-val, a polar-coordinate skeletal feature representation with anatomical constraints that enhances rotational invariance, and a multi-scale temporal modeling strategy that captures spatiotemporal dependencies efficiently through a two-layer GRU. The results show that the proposed framework provides high accuracy together with strong real-time performance, while reducing the parameter count by 33% relative to an LSTM-based solution, thereby offering a feasible approach for edge deployment.

The proposed method performs particularly well in underground power-grid working scenarios. It can accurately recognize workers’ postures and identify hazardous actions such as climbing and unsafe operations in a timely manner, thereby providing effective technical support for safety monitoring during power-grid maintenance. Through real-time monitoring and warning, the system has the potential to reduce operational risks substantially and support safer production management in the power industry. More broadly, the study shows that combining anatomical priors with lightweight deep learning is a practical route for biomimetic perception systems that seek to emulate human motion understanding in real-world environments.

### 5.2. Outlook

Future research will focus on several directions. First, multimodal information fusion will be investigated by combining RGB images with depth data and inertial measurement information to improve robustness in complex power-grid environments. Second, temporal modeling will be further optimized by exploring lightweight spatiotemporal feature extraction strategies and introducing richer dynamic cues beyond static-image recognition. In addition, self-supervised learning and domain adaptation will be studied to reduce dependence on large annotated datasets and improve generalization to unseen scenarios.

Further efforts will also be devoted to adapting the framework to more specific electric-power working procedures, such as grid inspection and equipment operation, and to developing an intelligent monitoring system for safety-oriented industrial production. The framework may also be extended to other high-risk industrial scenarios such as petrochemical plants and mining sites. These directions are expected to further promote the practical adoption of human action recognition technology in industrial safety applications.

## Figures and Tables

**Table 1:** Comparison of representative skeleton-based action recognition methods.

Method	Category	Key Feature	Complexity	Limitation
I3D [9]	CNN	RGB + temporal stream	High	Illumination sensitive
ST-GCN [2]	GCN	Spatial graph convolution	Medium	Fixed topology
2s-AGCN [14]	GCN	Adaptive graph convolution	High	Error accumulation
EfficientGCN [16]	GCN	Compound scaling	Medium	Complex architecture
PoseFormer [17]	Transformer	Spatial–temporal attention	O($T^{2}$)	High memory
PoseConv3D [19]	3D CNN	3D heatmap volumes	High	High memory
Proposed	RNN	Anatomical priors + polar encoding	Low (8.7 M)	Single-frame

**Table 2:** Experimental environment configuration.

Item	Configuration
CPU	Intel Xeon Gold 6248R (Intel Corporation, Santa Clara, CA, USA)
GPU	NVIDIA Tesla V100 (32 GB) × 4 (NVIDIA Corporation, Santa Clara, CA, USA)
Memory	256 GB DDR4
Operating system	Ubuntu 20.04 LTS (Canonical Group Limited, London, UK)
Deep learning framework	PyTorch 1.12.1 (Meta Platforms, Inc., Menlo Park, CA, USA)

**Table 3:** Results of the ablation study.

Model Configuration	Accuracy (%)	Macro-F1	Parameters (M)
Baseline (YOLO-Pose)	86.32	0.851	6.3
+ Polar-coordinate encoding	89.74	0.887	6.3
+ Adaptive keypoint filtering	92.15	0.912	6.5
+ Spatial attention mechanism	93.88	0.929	7.1
+ Multi-scale feature fusion	95.04	0.943	8.7
Full model	95.04	0.943	8.7

**Table 4:** Ablation study on training strategies on the SKPose test set.

Training Strategy	Acc. (%)	Macro-F1	Conv. (ep)	Gap
Single-stage end-to-end	88.7	0.874	80	6.2%
Two-stage (frozen pose backbone)	92.1	0.908	70	3.5%
Three-stage progressive (Ours)	95.04	0.943	60	2.1%
Curriculum learning	93.8	0.926	75	2.8%

**Table 5:** Ablation study on learning rate on the SKPose test set.

Learning Rate	Accuracy (%)	Macro-F1
$1\times 10^{-3}$	91.2	0.899
$1\times 10^{-4}$	94.1	0.931
$5\times 10^{-5}$	93.5	0.922
$3\times 10^{-4}$ (Ours)	**95.04**	**0.943**

**Table 6:** Ablation study on window size on the SKPose test set.

Window Size *L*	Accuracy (%)	Macro-F1
50	92.8	0.915
150	95.2	0.941
100 (Ours)	**95.04**	**0.943**

**Table 7:** Comparison with existing methods on the SKPose dataset.

Method	Accuracy (%)	Macro-F1	FPS	Params (M)
YOLO-Pose + SVM	82.15	0.806	12	8.2
YOLO-Pose + Random Forest	85.43	0.839	18	8.3
YOLO-Pose + MLP	88.92	0.874	25	8.5
ST-GCN	91.37	0.902	35	2.9
2s-AGCN	93.10	0.920	28	3.8
Proposed method	**95.04**	**0.943**	**48**	**8.7**

## Data Availability

The SKPose dataset used in this study is not publicly available due to proprietary restrictions imposed by China Southern Power Grid Company. Researchers wishing to access the data may contact the corresponding author with reasonable requests.
